# Molecular Mechanisms of Central Nervous System Axonal Regeneration and Remyelination: A Review

**DOI:** 10.3390/ijms21218116

**Published:** 2020-10-30

**Authors:** Akiko Uyeda, Rieko Muramatsu

**Affiliations:** Department of Molecular Pharmacology, National Institute of Neuroscience, National Center of Neurology and Psychiatry, Kodaira, Tokyo 187-8502, Japan; uyeda.a@ncnp.go.jp

**Keywords:** axon regeneration, sprouting, spinal cord injury, remyelination, BBB, systemic factor

## Abstract

Central nervous system (CNS) injury, including stroke, spinal cord injury, and traumatic brain injury, causes severe neurological symptoms such as sensory and motor deficits. Currently, there is no effective therapeutic method to restore neurological function because the adult CNS has limited capacity to regenerate after injury. Many efforts have been made to understand the molecular and cellular mechanisms underlying CNS regeneration and to establish novel therapeutic methods based on these mechanisms, with a variety of strategies including cell transplantation, modulation of cell intrinsic molecular mechanisms, and therapeutic targeting of the pathological nature of the extracellular environment in CNS injury. In this review, we will focus on the mechanisms that regulate CNS regeneration, highlighting the history, recent efforts, and questions left unanswered in this field.

## 1. Introduction

Damage to the central nervous system (CNS), such as in stroke, spinal cord injury (SCI), and traumatic brain injury, leads to severe neurological dysfunction due to neuronal cell death and axonal degeneration. Although neurons have the potential to undergo dynamic differentiation and development processes, their ability to regenerate after injury is very low in adults. Early studies have established that the pathogenic nature of the extracellular environment in CNS injury, such as myelin debris and glial scars, have inhibitory effects on axon regeneration [1,2]. In addition, emerging evidence has suggested that cell intrinsic mechanisms in adult CNS neurons also play an important role in the regenerative process after injury [3]. Considering that spontaneous axon regeneration is slightly observed in several non-primate and primate animal models of CNS injury [4,5] and is considered to contribute to partially restoring locomotor function, it should be worth focusing also on cell intrinsic mechanisms of CNS axon regeneration to promote this process. In addition to axonal regeneration, remyelination is also an important regenerative process to restore lost neurological function [6]. Although spontaneous remyelination often occurs in demyelinating diseases, which is mediated by oligodendrocytes, the mechanisms of this process are not as yet fully understood.

Recent advances in high-throughput technologies utilized in transcriptomics, proteomics, genomics, and cellular imaging have accelerated the identification of novel molecular mechanisms as a therapeutic target that effectively regulates axon growth, regeneration, and remyelination, and promotes functional recovery after CNS injury. Here, we review recent reports mainly focusing on the adaptation of novel techniques to investigate the molecular mechanisms regulating axonal regeneration and remyelination in the CNS.

## 2. Molecular Mechanisms of Axonal Regeneration

### 2.1. Role of Extrinsic Factors

**Figure 1:**
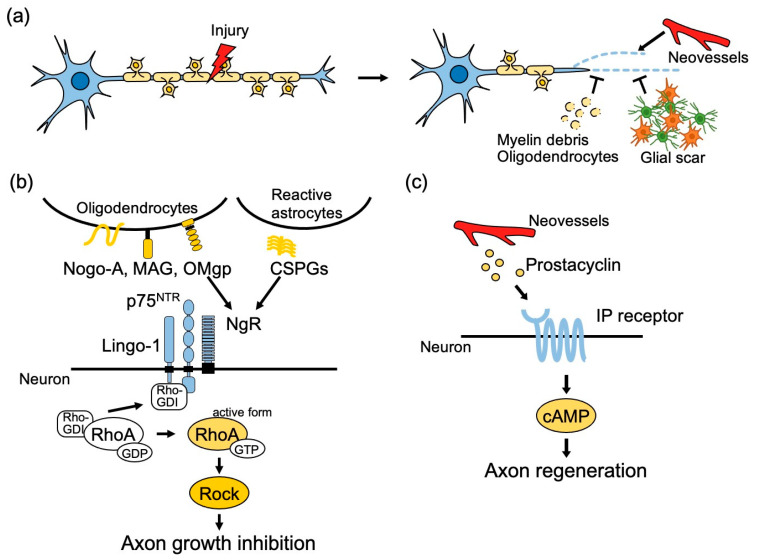
Molecular mechanisms underlying the microenvironmental regulation of axon regeneration in the adult central nervous system (CNS). (**a**) Illustration of the concept about inhibition of axon regeneration. (**b**) Myelin-associated glycoprotein (MAG), Nogo-A, and oligodendrocyte myelin glycoprotein (OMgp) expressed in oligodendrocytes and chondroitin sulfate proteoglycans (CSPGs) derived from reactive astrocytes around lesioned axons bind to Nogo receptor (NgR)/p75^NTR^/Lingo-1 receptor complex expressed in neurons and activate RhoA to inhibit axon growth. (**c**) CNS inflammation induces axonal degeneration, and partial rewiring of axons is followed by neovessel formation. Prostacyclin derived from endothelial cells binds to I type prostaglandin (IP) receptor expressed in the neurons, stimulates cyclic adenosine monophosphate (cAMP), and promotes axon regeneration.

In the mature CNS, neurons have little ability to regenerate their axons and reconstruct lost neural circuits after injury. It has been widely accepted that both extrinsic factors derived from the external environment of damaged axons and poor intrinsic potential for the regeneration of adult CNS neurons limit axonal regeneration and sprouting (Figure 1a). Growth repressive molecules secreted from glial scars around damaged areas, such as chondroitin sulfate proteoglycans (CSPGs), myelin-associated glycoprotein (MAG), Nogo, and oligodendrocyte-myelin glycoprotein (OMgp), have attracted much attention [7,8,9,10]. Preventing these extrinsic inhibitory signals has been one of the promising approaches to promote axon regeneration. These inhibitors bind to the Nogo receptor (NgR) expressed in neurons, which forms a receptor complex with p75^NTR^ and Lingo-1. p75^NTR^ mediates the activation of Ras homologous member A (RhoA), a Rho family small GTP-binding protein, by releasing Rho guanosine nucleotide dissociation inhibitors (Rho-GDIs) from GDP-bound inactive form RhoA. Then GTP-bound active form RhoA activates Rho kinase (ROCK), which leads to axon growth inhibition thorough phosphorylation of various molecules related to formation of cytoskeleton [11,12,13] (Figure 1b). C3 transferase, which blocks Rho activation by ADP-ribosylation, was found to promote the growth of corticospinal tract (CST) fibers and functional recovery after spinal cord injury (SCI) [14]. Other methods have been developed to improve specificity by RNA interference targeting RhoA. Adeno-associated viral (AAV)-mediated small hairpin RNA (shRNA) and poly(lactide-co-glycolide)-g-polyethylenimine-mediated small interfering (siRNA) delivery targeting RhoA improved axonal regeneration after optic nerve crush and SCI, respectively [15,16]. As another approach to exploring inhibitors of axonal growth, functional analyses of proteins that contribute to the formation of neural circuits during development have been performed to induce adult CNS regeneration. For example, the repulsive guidance molecule a (RGMa) is a glycosylphosphatidylinositol-anchored protein, identified as a repulsive factor that induces axonal outgrowth in the retina during development. RGM activates RhoA through Neogenin and Unc5B expressed in the neurons [17]. In addition, the transcription factor LMO4, which is thought to exert a potent inhibitory effect on axon growth, is also activated. Administration of RGMa-neutralizing antibodies to rats and rhesus monkeys after SCI has resulted in enhanced axonal regeneration and was observed to promote the recovery of motor function [18]. In addition, RGMa has been shown to be involved in neuroinflammation through immune system regulation [19,20]. It is expected to be effective in the treatment of various neurological diseases such as multiple sclerosis (MS) and Parkinson’s disease.

The lesion-specific environment not only inhibits axonal regeneration but, in many cases, also activates angiogenesis, which is thought to contribute to tissue repair, removal of inflammation, and elimination of ischemic damage [21]. A recent study revealed a link between angiogenesis and neuronal repair (Figure 1c). In the experimental autoimmune encephalomyelitis (EAE) model of MS, a substantial loss of CST axons causes neurological dysfunction, including motor deficits. This dysfunction partially recovers due to the spontaneous rewiring of remaining CST axons through sprouting, which is preceded by angiogenesis [22,23,24,25], raising the possibility that factors derived from newly formed vessels promote axonal regrowth. Indeed, the coculture of brain endothelial cells with cortical neurons in a transwell system was found to increase neurite length in a process mediated by prostacyclin secreted from endothelial cells [24]. Pharmacological screening leveled that prostacyclin enhances the synthesis of cyclic adenosine monophosphate (cAMP) in neurons through the I type prostaglandin receptor (IP receptor) [24,26]. IP receptor agonists enhanced CST sprouting and motor recovery in mice with EAE. When prostacyclin synthase (PGIS) was knocked down in endothelial cells by siRNA, the formation of CST sprouting in response to EAE decreased compared with that of the control, suggesting that prostacyclin from neovessels mediates axonal regeneration [24].

### 2.2. Role of Intrinsic Factors

In the last decade, advances in comprehensive transcriptome analysis have allowed effective identification of gene networks that regulate axon regeneration both negatively and positively. In many such studies, experimental animal models of spontaneous axon regeneration have been widely used (Table 1). For example, Cacna2d2 was newly identified as a gene that negatively regulates axon growth and regeneration based on comprehensive gene expression analysis using mouse dorsal root ganglion (DRG) neurons during development and after induction of regeneration by peripheral injury [27]. The Cacna2d2 gene encodes the α2δ2 subunit of membrane potential-dependent calcium channels (VGCCs), which regulates VGCC density at the membrane and the probability of vesicle release [28]. Cacna2d2 gene deletion and silencing promotes axonal growth of DRG neurons in vitro. Moreover, the pharmacological blockade of α2δ2 by systemic administration of pregabalin, which is clinically available as an anti-epileptic drug, was found to reduce synaptic transmission and promote axonal regeneration in spinal cord-injured mice in vivo [27]. Promoting axon sprouting from remaining intact fibers after CNS injury is thought to be one of the best effective strategies to restore lost neurological function. A recent study characterized the transcriptome of sprouting neurons from the CST of Nogo receptor 1 (Ngr1) knockout mice [29], which showed increased sprouting of intact CST after pyramidotomy compared with wild type mice [30]. In this study, a comparison between non-sprouting and sprouting CST neurons, which were identified by spinal retrograde labeling after pyramidotomy, was performed in wild-type and Ngr1 knockout mice. This analysis identified lysophosphatidic acid receptor 1 (Lpar1) as downregulated, and phospholipid phosphatase-related 1 (Lppr1) as upregulated genes in sprouting neurons, both of which are modulators of lysophosphatidic acid (LPA) signaling. LPPR1 overexpression in cultured cortical neurons enhances axon growth and regeneration. Moreover, in vivo viral overexpression of LPPR1 and pharmacological blockage of LPAR1 by systemic administration of AM095 were found to enhance CST sprouting and to promote functional recovery after pyramidotomy. Emerging evidence has confirmed that transplantation of spinal cord-derived neural progenitor cells (NPCs) enables robust regeneration of CST axons into the lesion site [31,32,33]. A recent study identified huntingtin (HTT) as a crucial transcriptional hub in the regenerative state of CST neurons derived from NPC grafts [34]. In that study, the cell type-specific ribosome pull-down method was used to extract actively translated mRNA from CST neurons of mice with SCI. Interestingly, injury alone triggered the regenerative state of the transcriptome, which was similar to gene expression in embryonic corticospinal neurons. NPCs grafts elongated this transcriptional reversal to an immature developing state and contributed to robust CST axon regeneration. Furthermore, HTT was found to be an upstream regulator of transcriptional change by bioinformatics technique. The beneficial effect of NPC grafts on axonal regeneration was diminished by the deletion of HTT in CST neurons [34].

Phenotypic screening of neuronal cells also emerged as a useful tool to identify new genes that regulate axon regeneration (Table 1). Inositol polyphosphate-5-phosphatase f (Inpp5f) was identified as a novel inhibitor of CNS axon regeneration using RNAi-mediated loss-of-function screen [35]. In this study, the screen was conducted with a focus on 219 phosphatases because of the limited number of genes encoding phosphatase in the mammalian genome, the relative possibility of developing small molecule compounds to block hit genes, and known examples of phosphatase that negatively regulate axon regeneration. Cultured mouse cortical neurons were treated with lentiviral particles containing shRNA, neurites were mechanically scraped by pins, and regenerating axons in the scraped zone were visualized by β-III tubulin staining after several days of culturing. Using this approach, 18 phosphatases, including phosphatase and tensin homolog (PTEN), were found to have a negative effect on axon regeneration. Among them, Inpp5f silencing led to the strongest increase in growth cone density as well as axon regrowth after injury. In addition, Inpp5f knockout mice showed enhanced CST axon sprout and recovery of motor function after mid-thoracic dorsal hemisection injury. More recently, target genes were expanded to 16,000 genome-wide protein-coding genes representing about 70% of the predicted protein-coding genes [36]. These genes, including those of Rab GTPases, were enriched in pathways related to intracellular transport. Inhibition of axon regeneration by Rab27 was confirmed in vivo using an axotomy model of *Caenorhabditis. elegans* lacking rab-27 in GABAergic DD/VD neurons. In addition, increased regeneration of the optic nerve was observed in Rab27 knockout mice after optic nerve crush. In this mouse, axon sprouting in raphespinal fibers was also increased, and functional recovery was improved after SCI, which was suggested to contribute to improved functional recovery.

As described above, repair of neural circuits requires genes involved in a wide range of intracellular mechanisms and continuous changes in gene expression. Epigenetic regulation has recently attracted much attention and has been suggested to be involved in this process. Micro RNAs (miRNAs) are a class of epigenetic regulation factors. It has been reported that a large number of miRNAs are differentially expressed after nervous system injury, suggesting that miRNAs play important roles in axon regeneration. A phenotypic screen using an miRNA library directly identified functional miRNAs in axon regeneration [37]. In this screening, the first screening was conducted with human SH-SY5Y cells, which were transduced with a lentiviral human genome-wide miRNA library containing thousands of miRNAs. Among these miRNAs, 13 miRNAs increased the neurite length of differentiated SH-SY5Y cells, and miR-135b had the largest effect. Mimics of miR-135b and its close homolog miR-135a increased axon growth of cultured hippocampal neurons by targeting and suppressing the expression of KLF4, an intrinsic inhibitor of axon growth and regeneration [38]. For in vivo, intravitreal injection of miR-135a and miR-135b mimics with lipofectamine enhanced regeneration of retinal ganglion cell (RGC) axon after optic nerve injury (ONI). In addition, RGC-specific overexpression of miR-135s by AAV2, a serotype known to be transduced RGCs, showed a cell-autonomous effect of miR-135s on axonal regeneration in RGCs. Considering that many miRNAs have been reported to target well-known inhibitors or activators of axon growth and regeneration, further investigation of these functions provides a therapeutic potential after CNS injury.

Screening using small molecules has also been utilized to investigate novel mechanisms underlying axonal regeneration [39,40,41]. A recent study performed the screening of 50,401 small molecules searching for compounds that promote axon growth in an inhibitory cellular environment mimicking the injured adult CNS [42]. Mouse embryonic stem cell-derived motor neurons expressing GFP [43] were cultured on CHO cells expressing MAG, and several rounds of screening on these neurons revealed that cholesterol-lowering drugs, including cerivastatin and simvastatin, most prominently enhanced neurite elongation. Statins inhibit 3-hydroxy-3-methylglutaryl-coenzyme A (HMG-CoA) reductase (HMGCR), which catalyzes cholesterol biosynthesis, and inhibition of protein prenylation is important for the stimulation of axon outgrowth downstream of HGCR inhibition by statins. Administration of cerivastatin and prenylation inhibitors was found to promote the axonal regeneration of RGCs after ONI. Furthermore, prenylation inhibitors also have strong axonal growth effects on motor neurons derived from human ES cells and human-induced pluripotent stem cells from an amyotrophic lateral sclerosis (ALS) patient, providing the therapeutic possibility for CNS regeneration [42].

## 3. Molecular Mechanism of Remyelination

### 3.1. Role of Extrinsic Factors

**Figure 2:**
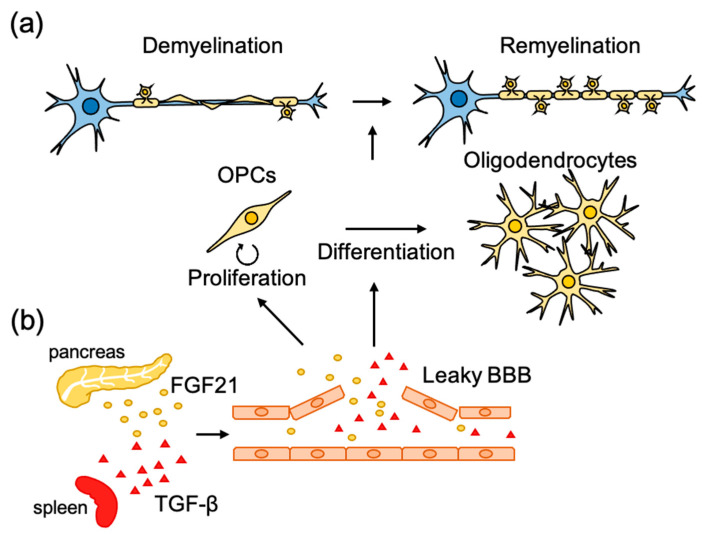
Molecular mechanisms underlying the microenvironmental regulation of remyelination. (**a**) Following injury to myelinated axons, remyelination is initiated with activation and recruitment of oligodendrocyte progenitor cells (OPCs) to the lesion sites. Then OPCs proliferate and differentiate to mature oligodendrocytes to form new myelin sheets. (**b**) Disruption of the blood–brain barrier (BBB) often occurs in CNS lesion sites. Systemic factors leaked from BBB also have beneficial effects on remyelination through promoting OPC proliferation and differentiation.

Demyelination in the CNS is one of the hallmarks of neurological disorders such as MS. Remyelination is a regenerative process that spontaneously occurs but is often insufficient to prevent axonal loss, restore lost axonal function, and improve neurological deficits after demyelination. Remyelination in the CNS is mainly mediated by adult oligodendrocyte progenitor cells (aOPCs), which are distributed throughout the mammalian CNS [44] (Figure 2a). aOPCs are considered to undergo several developmental phases during remyelination which are as follows: (i) Activation, in which aOPCs around the lesion site change their shape and gene expression profile; (ii) proliferation and migration, in which aOPCs increase their number and are recruited into the demyelinated area; (iii) differentiation, in which aOPCs differentiate into mature oligodendrocytes to form myelin sheaths [45,46,47]. In vitro models using cultured oligodendrocytes (either primary cells or derived from pluripotent stem cells) and in vivo models using lower vertebrates such as zebrafish have been used to study the biology of remyelination as well as myelination [48,49,50,51].

A lot of high-throughput screens using these models have provided several potential small molecules for remyelination, which have become a focus of clinical attention (Table 2) [52,53,54,55,56,57,58].

A small molecule screening using OPCs derived from rat optic nerve identified Benztropine, a muscarinic antagonist, as an inducer of OPC differentiation [52]. Benztropine-induced OPC differentiation was inhibited in the presence of carbachol, an agonist of muscarinic acetylcholine receptors (mAchRs), suggesting that benztropine promoted OPC differentiation through M1 mAchRs antagonism. Systemic administration of benztropine promoted functional recovery in EAE mice, a well-known model of MS, with increased remyelination capacity. In addition, benztropine treatment enhanced remyelination in a cuprizone-induced demyelination mouse model [52]. In a similar phenotypic screening using mouse epiblast stem cell-derived OPCs, 727 drugs that are safely used in clinical trials were tested for the maturation of OPCs into oligodendrocytes [54]. Twenty-two hit drugs were further investigated to determine whether they promote oligodendrocytes maturation in the CNS using cerebellar slices from postnatal mice, and miconazole and clobetasol were newly identified as inducers of oligodendrocyte maturation. The promotive effect of clobetasol treatment on OPC maturation was blocked by RU486, a glucocorticoid receptor antagonist, suggesting that glucocorticoid receptor signaling mediates OPC maturation downstream of clobetasol. For in vivo, systemic administration of clobetasol and miconazole enhanced the remyelination of the lysophosphatidylcholine (LPC)-induced demyelination model, in which focal demyelination is generated in the white matter of the dorsal spinal cord. Furthermore, both drugs were shown to have therapeutic effects in EAE mice by increasing expression of myelin basic protein (MBP) and reducing demyelination.

The next step after OPC differentiation is the formation of myelin sheets, but the cell-autonomous mechanisms regulating myelination in vitro have not been sufficiently investigated. Interestingly, oligodendrocytes can myelinate synthetic axon models, such as nanofibers. A recent study developed a platform with conical micropillar arrays to examine myelin formation around the 3D structure [53]. When OPCs were cultured and differentiated on this platform, both OPCs (stained with platelet-derived growth factor receptor-α (PDGFRα)) and oligodendrocytes (stained with MBP) interacted with pillars and formed rings around them, which were useful for high-throughput imaging and quantitative evaluation of oligodendrocyte proliferation, differentiation, and myelin formation. A total of 1000 bioactive molecules were tested using this platform, and 8 antimuscarinic compounds were found to enhance oligodendrocyte differentiation (increase MBP rings) but not proliferation (decrease PDGFRα rings). Among them, clemastine had the best effect on the differentiation and myelination of oligodendrocytes, and oral administration of clemastine after remyelination accelerated oligodendrocyte differentiation after LPC-induced demyelination [53]. Furthermore, it has been reported that clemastine also promotes remyelination and rescues behavioral changes in anxiety and cognitive function in a cuprizone-induced demyelination mouse model [59]. Screening of G-protein-coupled receptor targeting molecules was performed using the same platform [60], and κ-opioid receptor (KOR) agonists were identified, including U-50488, an enhancer of the differentiation and myelination of culture oligodendrocytes. Oral administration of U-50488 promoted remyelination after lysolecithin-induced demyelination in the corpus callosum, but had no effect on mice lacking KOR expression specifically in OPCs. These results suggest that KOR is a crucial regulator of oligodendrocyte differentiation and the target receptor for U-50488 during remyelination. Consistent with these findings, the opioid system has been suggested to be involved in the pathogenesis of MS [62,63], and another report showed the severe phenotype of EAE in KOR knockout mice [61]. In addition, U-50488 administration promoted remyelination in EAE and mice with cuprizone-induced demyelination. Pharmacological analysis with pathway inhibitors suggested the Gαi/o pathway, L-type calcium channels, or p38 pathway as downstream pathways that link KOR and the activation of oligodendrocyte differentiation [61].

### 3.2. Role of Systemic Factors

It has been reported that disruption of the vascular barrier often occurs in demyelinating diseases and related experimental animal models. Several circulating molecules in leaked blood have been shown to promote the development of OPCs during remyelination [64,65] (Figure 2b). In a pharmacological screening using an inhibitor library, fibroblast growth factor 21 (FGF21) in the serum was found to promote proliferation of cultured OPCs [64]. Injection of LPC into the spinal cord, which induces demyelination accompanied by disruption of the vascular barrier [66], was found to increase the concentration of FGF21 in the spinal cord at a similar time course as OPC proliferation in the lesion site. Spontaneous remyelination and neurological functional recovery after LPC injection were diminished in FGF21 knockout mice. FGF21 is highly expressed in the pancreas, and the promoting effect of serum on OPC proliferation became weak when FGF21 was specifically knocked down in the pancreas, suggesting that pancreas-derived FGF21 promotes OPC proliferation during remyelination [64]. The differentiation of OPCs into mature oligodendrocytes seems to be also regulated by systemic factors leaked by the disrupted vascular barrier. Through the use of pharmacological screening, transforming growth factor-β1 (TGF-β1) in the serum was found to promote the maturation of cultured oligodendrocytes [65] as assessed by extension of the MBP-positive area. Although the concentration of TGF-β1 in serum was higher than that in the cerebrospinal fluid, injection of LPC caused the accumulation of TGF-β1 at the lesion site. A reduction in circulating TGF-β1 by the depletion of platelets, which is known to be one of the main sources of circulating TGF-β1 [67,68,69], or by administration of TGF-β neutralizing antibody, was found to diminish spontaneous remyelination after LPC injection. Conversely, administration of TGF-β1 was found to promote remyelination in animal models of demyelination, cuprizone administration, and experimental autoimmune encephalomyelitis (EAE). These results suggest that the repair of the CNS is controlled by other biological systems, and it is hoped that research, which has been mainly limited to the brain and spinal cord, will find strategies to take into account the whole-body environment.

### 3.3. Role of Intrinsic Factors

A set of studies using comprehensive transcriptional analysis identified several key gene networks regulating remyelination. An earlier study identified a role for retinoid acid receptor RXR signaling in remyelination by transcriptome-profiling of rat cerebellar tissues, which were isolated at several time points after toxin-induced demyelination [70]. siRNA-mediated loss-of function of RXR-α and RXR-γ impaired the differentiation of cultured oligodendrocytes. Conversely, pharmacological activation of RXR by *9*-cis-retinoic acid (9cRA) promotes the differentiation of cultured oligodendrocytes. Intraperitoneally administration of 9cRA was found to promote remyelination after toxin-induced focal demyelination in rats. A recent study using mice expressing GFP in OPCs has provided more precise insight into the molecular mechanism regulating OPC differentiation in response to demyelinating injury [47]. In this analysis, GFP-expressing aOPCs were isolated from the corpus callosum of PDGFRα-GFP mice in which demyelination was induced by cuprizone feeding. Transcriptional analysis by microarray revealed that aOPCs change their gene expression in response to demyelination, which more closely resembles the observations in OPCs of neonatal animals. Functionally, aOPCs activated by demyelination had a greater migration rate and differentiation speed compared with those in the normal state. Biostatistical analysis of differentially expressed genes and in vitro validation suggested that increased expression of genes in the innate immune system, interleukin-1β (IL-1β), and CCL2 chemokine account for this functional change in aOPCs [47]. Another study described the transcriptional profile of oligodendrocyte lineage cells (OLCs) during remyelination, several weeks after cuprizone removal [71]. Olig1-cre mice were crossed with RiboTag mice [72] to generate mice expressing HA-tagged ribosomal protein in OLCs. These mice were fed with a cuprizone diet for 9 weeks, and OLC-specific ribosome-associated mRNAs were isolated from the corpus callosum with or without 3 weeks of remyelination with a normal diet. This comparison revealed the differences in gene expression profiles during demyelination and remyelination, and pathway analysis revealed that cholesterol synthesis pathways were enriched in upregulated pathways. Furthermore, estrogen receptor-β (ERβ) ligand (diarylpropionitrile) treatment directly enhanced the upregulation of cholesterol synthesis genes in oligodendrocytes. Web-available chromatin immunoprecipitation (ChIP)-seq data and ChIP assay on the N20.1 cell line, which has several characteristics of differentiating oligodendrocytes, suggested that ERβ ligand induced binding of ERβ to the transcription start sites of cholesterol synthesis genes. Cholesterol is an essential component of myelin [73], and it has been reported that dietary supplementation with cholesterol enhances remyelination after cuprizone-induced demyelination, which can cross the blood–brain barrier, as its permeability increases with cuprizone feeding [74]. These studies provide the therapeutic possibility for demyelinating disease by modulating cholesterol biogenesis in aOPCs or OLCs.

## 4. Conclusions

The ultimate goal of research on neural circuit repair is to reestablish functional neural circuits and restore lost neurological function. Recent advances in omics analysis and other high-content techniques have accelerated the understanding of the molecular mechanisms of axonal regeneration and remyelination, which is the first step toward that goal. Notably, there is a large amount of web-accessible resources and a number of data-mining software programs based on such studies, which allow us to effectively identify new therapeutic targets by reanalyzing data from different perspectives. However, it is still unclear how reconstruction of the circuitry restores motor and cognitive functions. The development of imaging and genetic modification techniques has made it possible to observe damaged neural circuits and analyze the function of specific genes. Continued investigation using these techniques will provide us with consistent knowledge from molecules to cells and neurological functions, which will contribute to the establishment of methods to re-acquire functional neural circuits.

## Figures and Tables

**Table 1:** Examples of current studies using omics to identify potential targets for axon regeneration.

Gene	Identify Method	In Vivo Manipulation/Outcomes	Reference
*Cacna2d2*	Transcriptome analysis of DRG neurons during development and regeneration	α2δ2 blockade by pregabalin/enhanced axon regeneration after SCI	[27]
*Lpar1*	Transcriptome analysis of CST sprouting neurons	LPAR1 blockage of LPAR1 by AM095/enhanced CST sprouting and functional recovery after pyramidotomy	[29]
*Lppr1*	Transcriptome analysis of CST sprouting neurons	AAV-mediated overexpression of LPPR1/enhanced CST sprouting and functional recovery after pyramidotomy	[29]
*huntingtin*	Transcriptome analysis of CST neurons after SCI with/without NPC grafts	HTT cKO in CST neurons/diminished CST axon regeneration after SCI with NPC grafts	[34]
*Inpp5f*	Functional genomic screening on cortical neurons	Inpp5f KO mice/enhanced CST sprouting and functional recovery after SCI	[35]
*Rab27*	Genome-wide functional genomic screening on cortical neurons	Rab27 KO mice/enhanced RGC regeneration after ONC, and enhanced RpST axon sprouting and functional recovery after SCI	[36]

DRG, dorsal root ganglion; CST, corticospinal tract; RpST, raphespinal tract; SCI, spinal cord injury; NPC, neural progenitor cell; cKO, conditional knock out; KO; knock out; RGC, retinal ganglion cell; ONC, optic nerve crush.

**Table 2:** Examples of studies using screening to identify potential therapy targets for remyelination.

Compound	Proposed Mechanism	Screening Model	In Vivo Model	Reference
Benztropine	Muscarinic receptor	OPCs from rat optic nerve	EAE and Cuprizone	[52]
Clemastine	Muscarinic receptor	OPCs from rat or mouse cortices	LPC, EAE, and Cuprizone	[53,59]
Miconazole	MAP kinase	Mouse ES-derived OPCs	LPC, EAE	[54]
Clobetasol	glucocorticoid receptor	Mouse ES-derived OPCs and mouse immortalized OL cell line	LPC, EAE, and NMO	[54,57,58]
U-50488	κ-opioid receptor	Mouse ESC-derived OPCs	LPC, EAE, and cuprizone	[60,61]

OPCs, oligodendrocyte progenitor cells; OL, oligodendrocyte; ES, embryonic stem cell; EAE, experimental autoimmune encephalomyelitis; LPC, lysophosphatidylcholine; NMO, neuromyelitis optica.

## Abbreviations

9cRA	RXR by 9-cis-retinoic acid
AAV	adeno-associated viral
aOPCs	adult oligodendrocyte progenitor cells
BBB	blood–brain barrier
cAMP	cyclic adenosine monophosphate
ChIP	chromatin immunoprecipitation
CNS	central nervous system
CSPGs	chondroitin sulfate proteoglycans
CST	corticospinal tract
DRG	dorsal root ganglion
EAE	autoimmune encephalomyelitis
ERβ	estrogen receptor-β
FGF21	fibroblast growth factor 21
HMGCR	3-hydroxy-3-methylglutaryl-coenzyme A (HMG-CoA) reductase
HTT	huntingtin
IL-1β	interleukin-1β
Inpp5f	inositol polyphosphate-5-phosphatase f
IP	I type prostaglandin
KOR	κ-opioid receptor
LPA	lysophosphatidic acid signaling
LPAR1	lysophosphatidic acid receptor 1
LPC	lysophosphatidylcholine
LPPR1	phospholipid phosphatase-related 1
MAG	myelin-associated glycoprotein
MBP	myelin basic protein
miRNAs	micro RNAs
MS	multiple sclerosis
Ngr1	Nogo receptor 1
NPCs	neural progenitor cells
OLCs	oligodendrocyte linage cells
OMgp	oligodendrocyte-myelin glycoprotein
ONI	optic nerve injury
PDGFRα	platelet-derived growth factor receptor-α
PGIS	prostacyclin synthase
PTEN	phosphatase and tensin homolog
RGC	retinal ganglion cell
RGMa	repulsive guidance molecule a
SCI	spinal cord injury
shRNA	small hairpin RNA
siRNA	small interfering
TGF-β1	transforming growth factor-β1
VGCCs	potential-dependent calcium channels
